# Feasibility and diagnostic validity of abdominal aortic aneurysm screening in Hungarian primary care: a multiregional pilot study

**DOI:** 10.3389/fmed.2026.1878337

**Published:** 2026-07-17

**Authors:** Róbert Kiss-Kovács, Ildikó Ambrus, Cintia Sipos, Szabolcs Fábián-Nagy, Györgyi Takács, Roland Tóth-Szeles, Erzsébet Kovács, Zsuzsanna Kaizer, Saddik Jdid Mahmoud, Renáta Ocskó-Horváth, Zsolt Palásthy, Albert Varga, Gergely Ágoston

**Affiliations:** 1 Department of Family Medicine, University of Szeged, Szeged, Hungary; 2 Primary Care Practice, Szatymaz, Hungary; 3 Primary Care Practice, Szeged, Hungary; 4 Primary Care Practice, Dóc, Hungary; 5 Primary Care Practice, Babót, Hungary; 6 Primary Care Practice, Pécel, Hungary; 7 Primary Care Practice, Ruzsa, Hungary; 8 Primary Care Practice, Hajdúböszörmény, Hungary; 9 Second Department of Internal Medicine and Cardiology Center, University of Szeged, Szeged, Hungary; 10 Department of Surgery, University of Szeged, Szeged, Hungary

**Keywords:** abdominal aortic aneurysm, diagnostic accuracy, feasibility, general practitioners, Hungary, point-of-care ultrasound, primary care, screening

## Abstract

**Background:**

Abdominal aortic aneurysm (AAA) is often asymptomatic but carries a high risk of mortality if rupture occurs. Several countries have implemented organized national screening programs to enable early detection of AAA, whereas no institutionalized screening program is currently available in Hungary. This multiregional pilot study aimed to assess the feasibility and diagnostic validity of AAA screening in Hungarian primary care.

**Methods:**

Following a structured theoretical and practical ultrasound (US) training program, 11 general practitioners (GPs) participated in a pilot AAA screening program. Screening examinations were performed in routine primary care settings, and all recorded US examinations underwent independent expert validation by two radiologists.

**Results:**

A total of 349 examinations were performed in 11 Hungarian primary care practices. Of these, 339 (97.1%) were considered interpretable after expert validation. Among the 339 participants with interpretable examinations, the mean age was 69.5 ± 3.1 years, 97.9% were male, and 22.4% were current smokers. Eight cases of AAA were identified, corresponding to a period prevalence of 2.36% (95% CI 1.02%–4.60%). The mean examination time was 6.6 ± 3.8 min. Agreement between GP-performed US and expert validation was substantial (κ = 0.772).

**Conclusion:**

Abdominal aortic aneurysm screening in Hungarian primary care appears feasible and shows high agreement with expert review when performed by trained GPs within a structured validation framework.

## 1 Introduction

Abdominal aortic aneurysm (AAA) is a relatively infrequent but potentially catastrophic vascular condition, characterized by a permanent dilatation of the abdominal aorta, conventionally defined as a maximal diameter of at least 3.0 cm or an increase of 1.5-fold compared with the adjacent normal segment (1, 2). The clinical importance of AAA arises from its largely asymptomatic natural course and the markedly high mortality associated with rupture, which remains one of the most lethal acute vascular emergencies despite advances in surgical and endovascular management. While elective repair of AAA is associated with perioperative mortality rates of approximately 2%–5%, mortality following rupture continues to exceed 30%–40%, underscoring the critical importance of early detection and preventive strategies (3).

Globally, AAA represents a relevant public health burden, with prevalence estimates of approximately 1% among individuals aged 30–79 years, corresponding to millions of affected individuals worldwide. Although AAA-related mortality has declined in recent decades, largely due to reductions in smoking prevalence and the implementation of screening programs in several high-income countries, the absolute number of individuals living with AAA continues to increase as a result of population aging (4). AAA occurs substantially more frequently in men than in women, while women with AAA exhibit a higher risk of rupture at smaller diameters and worse clinical outcomes (5). Cigarette smoking remains the most important modifiable risk factor, with additional contributions from advancing age, male sex, hypertension, hyperlipidemia, and family history (6–8).

The asymptomatic nature of most AAAs renders opportunistic detection unreliable, thereby providing a strong rationale for population-based screening. Over the past two decades, several countries have introduced organized AAA screening programs, most notably the National Health Service Abdominal Aortic Aneurysm Screening Programme (NAAASP) in England, which offers a one-time ultrasound (US) examination to men at the age of 65 years (9). Large randomized controlled trials, including the Multicenter Aneurysm Screening Study (MASS), have demonstrated that US-based screening significantly reduces AAA-related mortality through early detection and timely elective intervention (10–12). Consequently, major international organizations, including the United States Preventive Services Task Force (USPSTF) and European professional societies, recommend targeted AAA screening in defined high-risk populations (13, 14).

Despite this compelling evidence, the implementation of organized AAA screening remains heterogeneous across Europe. In several Central and Eastern European countries, including Hungary, no institutionalized nationwide screening program is currently available. Epidemiological estimates suggest that 1%–3% of Hungarian men over the age of 65 years have an abdominal aortic aneurysm, while national data indicate persistently high rupture-related mortality and comparatively low rates of elective AAA repair compared with countries with established screening systems (15, 16). This discrepancy highlights a substantial gap between evidence-based recommendations and real-world clinical practice.

In parallel, the diagnostic landscape of primary care has evolved considerably with the increasing availability of point-of-care ultrasound (PoCUS) devices and structured training programs for general practitioners (GPs) (17, 18). US represents the imaging modality of choice for AAA screening, offering high diagnostic accuracy comparable to computed tomography, while avoiding ionizing radiation, contrast exposure, and high costs (19). Importantly, accumulating evidence indicates that, following appropriate training, non-radiologist clinicians, including GPs, are capable of performing focused US examinations with high sensitivity and specificity for AAA detection (20–22). These developments have led to growing interest in decentralized, primary care-based screening approaches.

General practitioners, as first-contact providers, may be well positioned to identify and access high-risk individuals, particularly in settings where organized screening programs are lacking. However, robust evidence regarding the feasibility and diagnostic reliability of GP-performed AAA screening in real-world primary care settings remains limited, especially in Central and Eastern Europe (23, 24). In particular, few studies have incorporated systematic external validation of GP-acquired US examinations, which is essential for ensuring diagnostic safety and reproducibility (21, 22). Against this background, the present multiregional pilot study aimed to assess the feasibility and diagnostic validity of AAA screening performed by trained GPs using PoCUS in Hungarian primary care. By integrating structured training, standardized imaging protocols, and independent expert validation, this study seeks to provide clinically relevant evidence on the practicality and reliability of GP-led AAA screening and to inform the potential development of future screening strategies.

## 2 Materials and methods

### 2.1 Study design and setting

**FIGURE 1:**
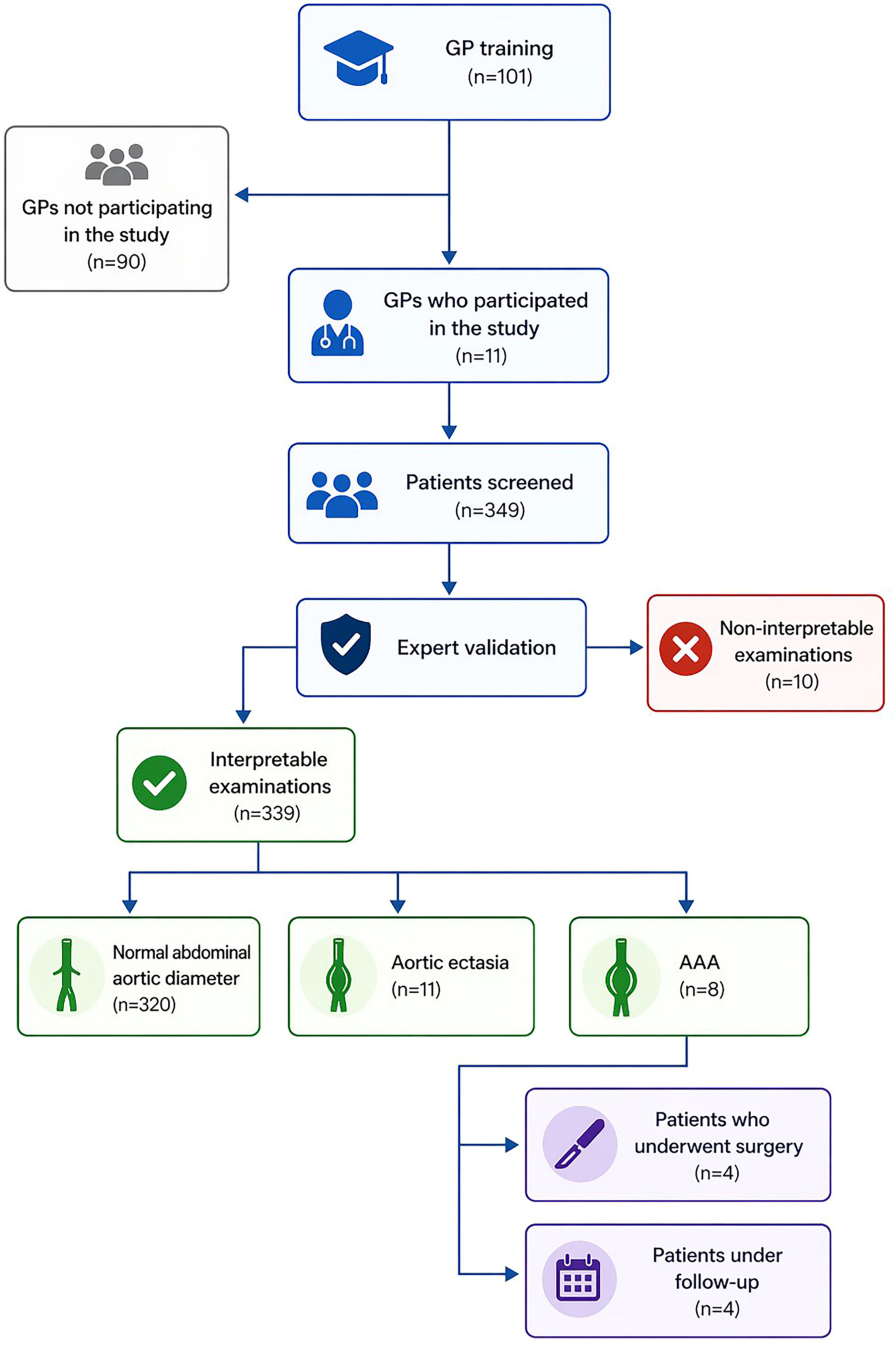
Study flowchart of GP participation, patient screening, and outcomes of AAA screening examinations. After training, a subset of GPs contributed to patient recruitment and US assessments. The majority of examinations were suitable for analysis following expert validation. Most patients demonstrated normal abdominal aortic findings, while a smaller proportion showed pathological changes, including ectasia and aneurysm. Abdominal aortic ectasia was defined as an aortic diameter of 25.0–29.9 mm. Cases identified with aneurysmal dilation were subsequently managed either by referral for surgical intervention or by scheduled follow-up. AAA, abdominal aortic aneurysm; GP, general practitioner.

This multicenter, multiregional pilot validation study used a cross-sectional design to evaluate the feasibility and diagnostic reliability of AAA screening performed by GPs using PoCUS in Hungarian primary care. The study was conducted under routine clinical conditions to reflect real-world primary care practice, while ensuring diagnostic safety through structured external validation of all US examinations. The study followed a predefined training-screening-validation-referral framework, as presented in Figure 1. After completing a standardized training program, participating GPs performed abdominal aortic US examinations during routine patient care, with mandatory digital documentation of a predefined set of images. All examinations were subsequently submitted for blinded expert review by experienced radiologists. In cases of suspected or confirmed AAA, patients were referred according to a predefined clinical pathway for further diagnostic evaluation and specialist management. Screening activities were conducted in primary care practices across six Hungarian counties, ensuring broad geographic representation. Academic coordination and methodological oversight were provided by the Departments of Family Medicine, Radiology, and Surgery at the University of Szeged, in collaboration with participating primary care practices. US images were stored and transferred via a secure digital platform, enabling centralized validation and structured feedback on image quality and measurement accuracy.

### 2.2 Participants

Participant recruitment was conducted between September 2024 and December 2025 using a combination of targeted invitations and opportunistic enrollment during routine visits to participating primary care practices. Prior to inclusion, all participants received detailed verbal and written information about the study, and written informed consent was obtained. A total of 349 individuals underwent AAA screening during the study period. Eligible participants included men aged 65–75 years regardless of smoking status, as well as men and women aged ≥ 50 years with either a personal history or a first-degree family history of aneurysmal disease in accordance with international screening recommendations and US practice guidelines (13, 23). Exclusion criteria included conditions associated with limited life expectancy or high perioperative risk, as well as any condition that impaired the ability to provide informed consent or comply with study procedures.

### 2.3 Training of GPs

Prior to the initiation of screening activities, 101 GPs completed a standardized 2-day accredited US training program designed to support the implementation of AAA screening in primary care. The program combined theoretical instruction with hands-on practical training under the supervision of experienced radiologists. The theoretical component covered abdominal aortic anatomy, indications and limitations of AAA screening, and principles of accurate measurement, while the practical component focused on standardized image acquisition and appropriate use of PoCUS devices. Following training, participation in the screening program was voluntary. A total of 25 trained GPs registered for participation, of whom 11 performed screening examinations included in the present analysis across six Hungarian counties. Competency was assessed at the end of the training program through supervised examinations, and only GPs who demonstrated adequate technical and interpretative proficiency across three consecutive patient assessments were authorized to perform independent screening examinations. Ongoing quality assurance was ensured through centralized expert validation and structured feedback throughout the study period.

### 2.4 Screening procedure

Ultrasound examinations were performed in accordance with the 2025 AIUM practice parameter for diagnostic and screening ultrasound of the abdominal aorta in adults (25). The abdominal aorta was systematically examined from the level of the diaphragm to the aortic bifurcation. According to the study protocol, three labeled transverse still images were recorded in each participant, corresponding to the proximal, mid, and distal abdominal aortic segments. At each of these three levels, both anteroposterior and transverse diameters were measured using the outer-to-outer caliper technique, with care taken to obtain measurements perpendicular to the long axis of the vessel. In addition, at least one longitudinal still image or cine clip of the abdominal aorta was recorded to document the course of the vessel and to support anatomical orientation.

Thus, in participants with normal aortic findings, the standard minimum documentation set consisted of four recorded ultrasound items: three transverse still images with visible caliper measurements and one longitudinal still image or cine clip. When ectasia or suspected aneurysmal dilatation was identified, additional documentation was required by the protocol. In these cases, further transverse images were recorded at the site of maximal aortic diameter, and additional longitudinal images and/or cine clips were obtained to document the longitudinal extent of the dilatation and its anatomical relationship to the renal arteries and the aortic bifurcation. Consequently, examinations with ectasia or suspected AAA included at least six recorded ultrasound items, with further images or cine clips added when necessary to ensure complete documentation of maximal diameter, aneurysm extent, and anatomical localization.

For diagnostic classification, the larger of the anteroposterior and transverse outer-to-outer diameters was used. Aortic diameters were categorized as normal (<25.0 mm), ectasia (25.0–29.9 mm), and abdominal aortic aneurysm (≥30.0 mm). AAA was defined as a maximal aortic diameter ≥ 3.0 cm or a ≥1.5-fold increase compared with the adjacent non-dilated segment (1). All examinations were performed using commercially available ultrasound devices (GE Vscan Air, GE Healthcare, Chicago, IL, USA; and Mindray M6, Mindray, Shenzhen, China) equipped with convex probes in participating primary care practices. Examination duration was recorded as part of the feasibility assessment.

### 2.5 Validation of examinations

All recorded US examinations were independently reviewed by two experienced radiologists who were blinded to participant clinical characteristics and to the initial GP interpretation. The validation process followed a structured protocol assessing image quality, completeness of the predefined image set, measurement accuracy, and diagnostic classification. Each examination was classified as interpretable or non-interpretable based on predefined quality criteria. Interpretable examinations were further categorized according to aortic findings, including normal aortic diameter, ectasia, or AAA. Examinations deemed non-interpretable due to inadequate image quality or incomplete documentation were recorded as such, and patients were advised to undergo further diagnostic evaluation when clinically indicated. This approach ensured rigorous diagnostic validation while minimizing observer-related bias. Expert validation was based exclusively on the still images and cine clips acquired and submitted by the GPs. The reviewing radiologists did not perform independent ultrasound examinations, and an independent confirmatory imaging examination, such as radiologist-performed ultrasound or CT angiography, was not systematically obtained for all participants. The validation process therefore assessed the quality, completeness, measurements, and diagnostic classification of the submitted image sets rather than providing a fully independent participant-level reference examination of the entire abdominal aorta.

### 2.6 Statistical analysis

Statistical analyses were performed using IBM^®^ SPSS^®^ Statistics version 29. Continuous variables were expressed as mean ± standard deviation (SD) or median (interquartile range, IQR), depending on data distribution, while categorical variables were presented as frequencies and percentages. Normality of continuous variables was assessed using the Shapiro-Wilk test. As most variables deviated from a normal distribution, non-parametric methods were applied. Comparisons between groups were performed using the non-parametric Mann-Whitney U test. Categorical variables were compared using Fisher’s exact test due to small expected cell counts. The prevalence of AAA was calculated for the overall population and key subgroups, and presented with 95% confidence intervals (CI). Agreement between GP-performed US and expert validation was assessed using Cohen’s kappa (κ) statistic, with overall percentage agreement also reported. Feasibility outcomes were assessed by analyzing examination duration and the proportion of non-interpretable examinations. Due to the limited number of AAA cases, multivariable analysis was not performed. A two-sided *p*-value < 0.05 was considered statistically significant. Using expert validation of the submitted image set as the comparator, sensitivity, specificity, positive predictive value (PPV), and negative predictive value (NPV) were calculated with exact binomial 95% confidence intervals.

## 3 Results

### 3.1 Characteristics of the screened population

A total of 349 individuals underwent AAA screening, of whom 339 examinations were deemed evaluable following expert validation. The cohort was predominantly male and reflected a high-risk screening population, with a substantial proportion of participants reporting current or previous smoking exposure. Women were eligible for screening only if they were aged ≥ 50 years and had a personal or first-degree family history of aneurysmal disease; therefore, women constituted a selected higher-risk subgroup rather than a general screening population. Seven women were included in the interpretable cohort, representing 2.1% of the study population. Baseline characteristics of the screened population are summarized in Table 1.

**TABLE 1:** Baseline characteristics of patients with interpretable examinations according to AAA status.

Variable	Total (*n* = 339)	AAA (*n* = 8)	Non-AAA (*n* = 331)	*P*-value
Age, years	69.5 ± 3.1	71.5 ± 4.1	69.5 ± 3.1	0.079
Male sex, *n* (%)	332 (97.9)	7 (87.5)	325 (98.2)	0.155
Ever smoked ≥ 100 cigarettes, *n* (%)	150 (44.2)	5 (62.5)	145 (43.8)	0.474
Current smoker, *n* (%)	76 (22.4)	4 (50.0)	72 (21.8)	0.079
Family history of peripheral aneurysmal vascular disease, *n* (%)	9 (2.7)	2 (25.0)	7 (2.1)	0.016
Personal history of peripheral aneurysmal vascular disease, *n* (%)	4 (1.2)	1 (12.5)	3 (0.9)	0.091
History of aortic surgery, *n* (%)	1 (0.3)	0 (0.0)	1 (0.3)	1.000
Screening time, min	6.6 ± 3.8	12.3 ± 3.5	6.5 ± 3.8	<0.001

Values are presented as mean ± standard deviation (SD), median (interquartile range, IQR), or number (percentage), as appropriate. Comparisons between groups (AAA vs. non-AAA) were performed using the Mann-Whitney U test for continuous variables and Fisher’s exact test for categorical variables, due to the small number of AAA cases.

Values are presented as mean ± standard deviation (SD), median (interquartile range, IQR), or number (percentage), as appropriate. Comparisons between groups (AAA vs. non-AAA) were performed using the Mann-Whitney U test for continuous variables and Fisher’s exact test for categorical variables, due to the small number of AAA cases.

### 3.2 Feasibility of GP-led AAA screening

**FIGURE 2:**
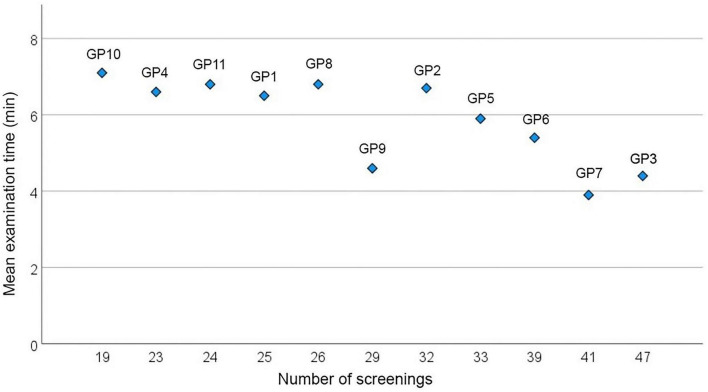
Relationship between screening volume and mean examination time among GPs. Each point represents an individual GP. In this exploratory operator-level analysis, a strong negative correlation was observed between the number of screening examinations performed and mean examination time, suggesting shorter examination times with increasing operator experience. Because the analysis included only 11 GPs, this finding should be interpreted cautiously and considered hypothesis-generating.

A total of 349 AAA US screening examinations were performed by GPs, of which 339 (97.1%) were considered interpretable following expert validation. Ten examinations (2.9%) were classified as non-interpretable because an adequate acoustic window could not be obtained. Qualitative assessment during the GP examinations and subsequent expert review of the stored images indicated that increased abdominal soft-tissue depth and extensive bowel gas were the principal factors limiting adequate visualization of the abdominal aorta. The mean examination time was 6.6 ± 3.8 min, with individual screening times ranging from 2.1 to 11.6 min. As presented in Figure 2, an exploratory operator-level analysis showed a strong negative correlation was observed between the number of examinations performed by individual GPs and their mean examination time (*r* = −0.811, *p* = 0.002), suggesting shorter examination times among GPs who performed a higher number of examinations.

### 3.3 Prevalence and characteristics of AAA

**FIGURE 3:**
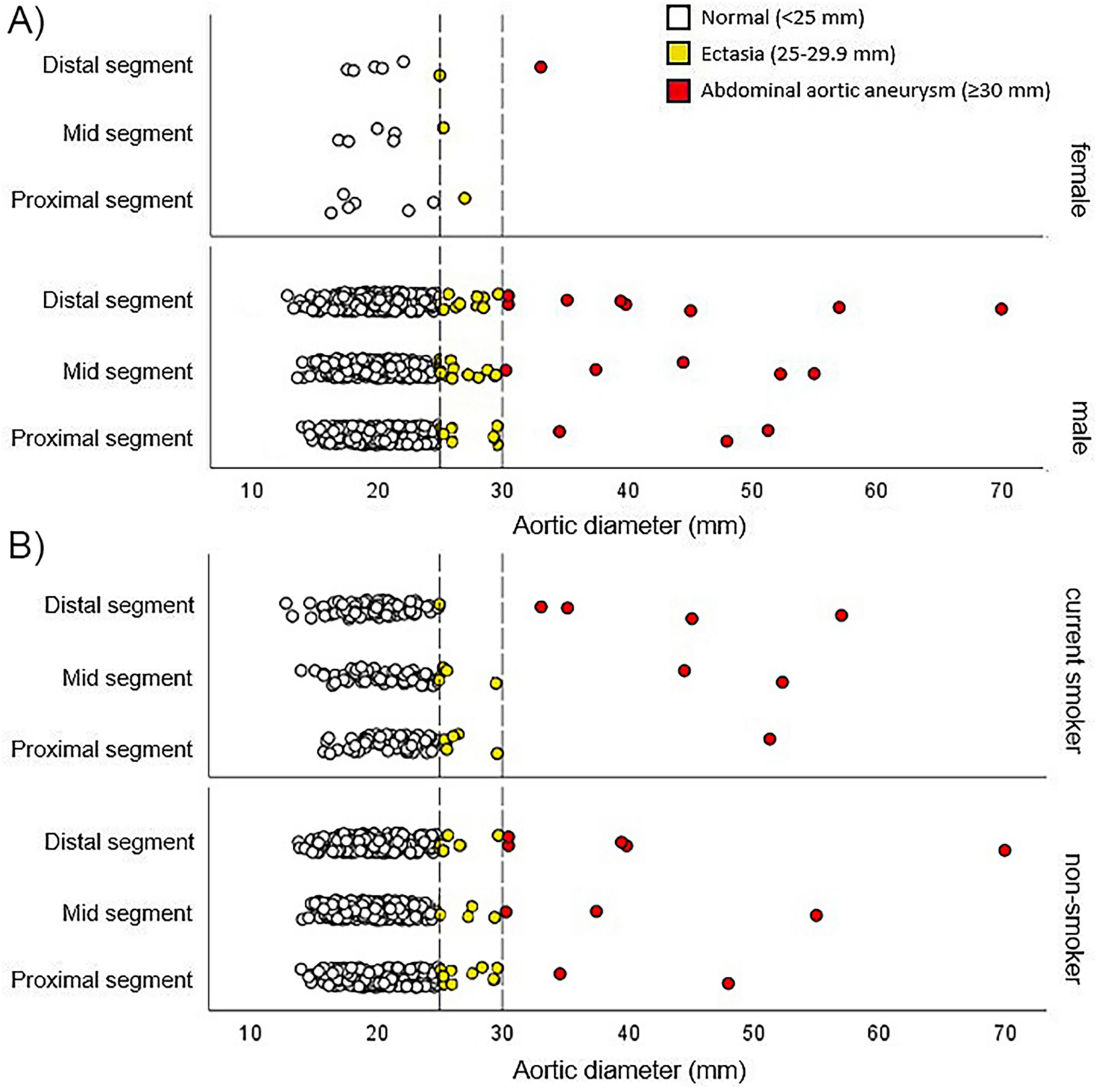
Distribution of abdominal aortic diameters across aortic segments stratified by sex and smoking status. **(A)** (Upper) shows the distribution of segmental abdominal aortic diameters stratified by sex, while **(B)** (Lower) presents the same distribution stratified by smoking status. Within each panel, measurements are grouped by aortic segment (proximal, mid, and distal). Each point represents an individual segmental measurement obtained during ultrasound screening performed by GPs. Points are color-coded according to aortic diameter category: normal (<25 mm), ectasia (25–29.9 mm), and abdominal aortic aneurysm (≥30 mm). Vertical dashed lines indicate clinically relevant threshold values for ectasia and aneurysm. For each segment, the larger of the anteroposterior and transverse diameters was used for analysis. Normal aortic measurements are shown as white markers with black borders, ectasia is shown with yellow markers, and abdominal aortic aneurysm is shown with red markers.

Abdominal aortic aneurysm was identified in 8 of 339 evaluable participants, corresponding to a period prevalence of 2.36% (95% CI 1.02–4.60). Among individuals with AAA, 7 were male and 1 was female. Regarding smoking status, 5 participants had a history of smoking, of whom 4 were current smokers, while 3 had never smoked. The segmental distribution of aortic diameters stratified by sex and smoking status is shown in Figure 3, demonstrating that aneurysms were predominantly observed in the distal aortic segment. Of the eight participants with expert-confirmed AAA, four underwent vascular surgical intervention and four were managed by imaging surveillance. Among those who underwent surgery, the validated maximal transverse AAA diameters were 55 mm, 57 mm, 57 mm, and 70 mm, respectively. Among participants managed by surveillance, the corresponding validated maximal transverse diameters were 40 mm, 45 mm, 46 mm, and 48 mm.

### 3.4 Comparison between participants with and without AAA

Comparative analyses between participants with and without AAA are presented in Table 1. Participants with AAA tended to be older and were more frequently current smokers than those without AAA; however, these differences did not reach statistical significance. In contrast, a positive family history of aneurysmal vascular disease was significantly more frequent among participants with AAA. Screening time was also significantly longer in the AAA group, which likely reflects the need for more detailed assessment and documentation in cases with pathological aortic dilatation.

### 3.5 Agreement between GP and expert validation

Agreement between GP-performed AAA screening and radiologist validation was assessed among the 339 interpretable examinations. GP classification agreed with expert validation in 335 cases (98.8%). Cohen’s kappa indicated substantial agreement regarding the presence of AAA (κ = 0.772, *p* < 0.001). However, this estimate should be interpreted in the context of the low prevalence of AAA and the marked imbalance between positive and negative cases.

Diagnostic classification yielded 7 true-positive, 328 true-negative, 3 false-positive, and 1 false-negative result. Using expert validation of the submitted image set as the comparator, sensitivity was 87.5% (95% CI 47.3%–99.7%), specificity was 99.1% (95% CI 97.4%–99.8%), positive predictive value was 70.0% (95% CI 34.8%–93.3%), and negative predictive value was 99.7% (95% CI 98.3%–100.0%).

To further characterize discordant classifications, the three false-positive and one false-negative cases were reviewed at case level. The three false-positive classifications occurred in cases with GP-measured maximal abdominal aortic diameters of 32 mm, 34 mm, and 31 mm, respectively, whereas the corresponding expert-validated maximal diameters were 29 mm, 28 mm, and 29 mm. All three GP measurements therefore slightly exceeded the AAA threshold, whereas expert validation classified the same examinations as non-aneurysmal. Review of the stored images suggested overestimation of the maximal transverse diameter due to technical measurement factors, including slightly oblique transverse sectioning, incomplete perpendicular alignment to the long axis of the abdominal aorta, lateral-wall acoustic shadowing, and/or caliper placement at the vessel margin.

The single false-negative case was also classified as interpretable. In this case, the GP-measured maximal diameter was 26 mm, whereas expert validation identified a maximal aortic diameter of 32 mm, meeting the diagnostic threshold for AAA.

## 4 Discussion

### 4.1 Main findings

In this multiregional primary care-based pilot study, AAA screening performed by trained GPs using PoCUS was feasible and showed high agreement with expert review of submitted image sets. Feasibility was supported by a high proportion of interpretable examinations and a short mean examination time, compatible with routine primary care workflows. An exploratory operator-level signal suggested improved efficiency with increasing screening volume. The observed AAA prevalence was consistent with estimates from comparable high-risk screening cohorts, and family history of peripheral aneurysmal vascular disease was more frequent among participants with AAA.

### 4.2 Comparison with the literature

The findings of the present study are in line with a growing body of evidence supporting the effectiveness of AAA screening and extend these observations to a primary care setting using GP-performed PoCUS. The clinical benefit of AAA screening has been established in large population-based studies and long-term follow-up analyses, which consistently demonstrate reductions in AAA-related mortality through early detection and elective intervention (26–28). However, organized screening programs remain inconsistently available across Europe, particularly in Central and Eastern European regions, where opportunistic or decentralized screening strategies may represent a feasible complementary approach (23, 28).

The high feasibility observed in our study, reflected by a 97.1% interpretability rate and short examination times, is consistent with previous studies showing that focused ultrasound examinations can be incorporated into routine clinical workflows (24, 29, 30). The observed examination time was comparable to, or lower than, that reported in previous primary care AAA screening studies (31). The exploratory operator-level finding of shorter examination times with increasing screening volume is also consistent with literature showing that ultrasound performance improves with structured training and practice (32, 33).

The level of agreement between GP-performed ultrasound and expert validation in the present study is comparable to previous reports involving non-radiologist ultrasound operators. Prior studies have shown that, after appropriate training, non-radiologist clinicians can achieve high diagnostic accuracy for AAA detection, with sensitivity and specificity approaching those of formal imaging modalities (31, 33, 34).

The observed AAA prevalence of 2.36% is consistent with contemporary epidemiological data from screening cohorts, which report prevalence rates of approximately 1%–3% among older men in high-risk populations (5, 35). The associations observed between AAA and established risk factors, particularly smoking and family history, are also consistent with the recognized multifactorial pathogenesis of AAA (36, 37).

### 4.3 Implications for research, clinical practice, education and policy

The findings of the present study have implications for clinical practice, medical education, research, and healthcare policy, particularly within Hungarian primary care and similar Central and Eastern European healthcare systems.

From a clinical perspective, GP-led AAA screening using PoCUS may provide a feasible approach to identifying high-risk individuals in settings where organized nationwide screening is not available. In Hungary, this strategy could support earlier detection, timely referral, and specialist assessment of patients with suspected aneurysmal dilatation (38, 39).

From an educational and implementation perspective, broader adoption would require more than initial ultrasound training alone. Competency-based training, supervised practice, predefined certification criteria, periodic review of stored examinations, individual feedback, and refresher training would be necessary to maintain quality across practices (40, 41). Access to expert consultation for technically limited, borderline, or abnormal examinations, together with standardized documentation requirements and clearly defined referral pathways, would also be required to support diagnostic safety.

From a research perspective, larger multicenter studies are needed to evaluate diagnostic performance, cost-effectiveness, patient acceptance, adherence to follow-up, and long-term clinical outcomes of GP-led AAA screening in Hungary. Future investigations should also examine how AAA screening could be integrated with existing cardiovascular risk assessment strategies in primary care (42, 43).

From a health policy perspective, decentralized primary care-based screening may represent a complementary strategy in healthcare systems where organized screening programs are not yet established (44). In regions with limited access to specialized imaging, integrating PoCUS into primary care could improve equity in access to diagnostic services and enable more efficient referral of patients with suspected AAA (45, 46).

### 4.4 Strengths and limitations

This study has several important strengths. It was conducted in real-world primary care settings across six Hungarian counties and applied a structured training-screening-validation-referral framework. All submitted ultrasound examinations underwent blinded expert review by two experienced radiologists, providing systematic external quality control. The study also provides novel data from a Central and Eastern European setting, where evidence on AAA screening in primary care remains limited.

Several limitations should also be acknowledged. First, the study included a relatively small number of expert-confirmed AAA cases, which limited statistical power and the interpretation of subgroup comparisons. The low number of AAA-positive cases also resulted in wide confidence intervals around sensitivity and positive predictive value; therefore, the reported diagnostic performance measures should be considered descriptive pilot estimates rather than definitive evidence of diagnostic accuracy.

Second, potential selection bias at the GP level may limit generalizability. Participation was voluntary, and GPs who enrolled in the study and completed competency assessment likely represented a particularly motivated and technically capable subgroup. The high interpretability rate and observed diagnostic agreement may therefore overestimate performance achievable in broader implementation. Similarly, the operator-level learning curve analysis included only 11 GPs and should be considered hypothesis-generating.

Third, BMI, waist circumference, and abdominal fat thickness were not collected or measured. Although increased abdominal soft-tissue depth and extensive bowel gas were noted qualitatively during GP examinations and expert review, the relationship between body habitus and examination interpretability could not be quantified.

Fourth, the expert validation framework should not be interpreted as a fully independent participant-level reference standard. Radiologists reviewed the still images and cine clips acquired and submitted by GPs, and independent radiologist-performed ultrasound or CT angiography was not systematically obtained for all participants. Expert assessment was therefore constrained by the anatomical coverage, image quality, and documentation selected by the GP operator. Accordingly, the reported agreement and diagnostic performance measures describe GP interpretation relative to expert review of submitted image sets and cannot establish true participant-level sensitivity for the entire abdominal aorta.

Finally, the cross-sectional design precludes assessment of long-term clinical outcomes, adherence to surveillance, and the downstream clinical impact of GP-led AAA screening.

### 4.5 Overall conclusion

This study demonstrates that AAA screening performed by trained GPs using PoCUS is feasible, time-efficient, and shows high agreement with expert review of submitted image sets in routine primary care. In the absence of an established national screening program, GP-led ultrasound screening may represent a practical approach to improving early detection of AAA in Hungary and similar healthcare systems when embedded in a structured quality framework.

## Data Availability

The original contributions presented in this study are included in the article/supplementary material, further inquiries can be directed to the corresponding author.
